# Intracellular Regulation of Cross-Presentation during Dendritic Cell Maturation

**DOI:** 10.1371/journal.pone.0076801

**Published:** 2013-10-03

**Authors:** Claudia S. Wagner, Jeff Grotzke, Peter Cresswell

**Affiliations:** Department of Immunobiology, Howard Hughes Medical Institute, Yale University School of Medicine, New Haven, Connecticut, United States of America; Oklahoma Medical Research Foundation, United States of America

## Abstract

We have investigated the effect of different maturation stimuli on the ability of mature dendritic cells (DCs) to cross-present newly acquired particulate antigens. Cross-presentation was impaired in DCs matured by treatment with TNF-α, CpG and LPS, but was less affected upon CD40L-induced maturation. The difference could not be explained by decreased antigen uptake or translocation into the cytosol, but decreased cross-presentation ability did correlate with increased phagosomal/lysosomal acidification. Nevertheless, intra-phagosomal degradation of OVA was not increased in matured samples, suggesting that decreasing phagosomal pH may also regulate cross-presentation by a mechanism other than enhancing degradation.

## Introduction

DCs are remarkably efficient at cross-presentation, a process that allows exogenous antigens to be presented by MHC class I (MHC I) molecules to CD8-positive T cells. Although a number of different pathways have been identified, cross-presentation of most antigens depends on MHC I association with peptides that are generated by the proteasome in the cytosol. From there the peptides are either transported into the ER, as they are for conventional MHC I loading, or back into phagosomal/endosomal compartments that contain critical components derived from the ER, including the Transporter associated with Antigen Processing (TAP) [1,2].

A variety of stimuli, including microbial products, interaction with other cells, mediators of tissue damage, or inflammatory cytokines, cause DCs to undergo a maturation process that is required for the initiation of immune responses. Many cellular functions change during that process, such as motility, expression of surface co-stimulatory molecules, cytokine production and, important for this study, the capacity for antigen presentation [3–5]. MHC class II (MHC II)-restricted antigen presentation is dramatically affected by DC maturation, and the mechanisms that regulate this have been well-studied [5]. However, less is known concerning the effects of maturation on cross-presentation.

Depending on the timing of antigen capture, maturation may differently impact the process of cross-presentation. In case of maturation that occurs simultaneously with or shortly after antigen capture, certain stimuli were found to enhance cross-presentation; these include lipopolysaccharide (LPS) [6,7], immune complexes [8,9], disruption of cell-cell contact or CD40L stimulation [10]. However, CPG [10,11], low dose LPS, Poly(I:C) or TNF-α [10] did not promote cross-presentation. In case of antigen capture that takes place in already mature DCs, CpG, LPS, or poly (I:C) either failed to affect or enhanced cross-presentation [9,11–16]. On the other hand, cross-presentation may also be inhibited by peptidoglycan and other TLR ligands [6,12,17,18]. Of note, soluble ovalbumin (OVA), used in several studies, needs an additional maturation stimulus after antigen capture for cross-presentation to occur efficiently [6,10].

Mechanistic explanations for regulation of cross-presentation during DC maturation are limited. Decreased antigen uptake [6,12,17] and inhibition of antigen access to the cytosol have been proposed to explain inhibition of cross-presentation in mature DCs [6].

Maturation-induced signaling pathways may modulate cross-presentation through alterations of intra-phagosomal antigen routing and/or degradation, given that TLR-mediated DC activation enhances lysosomal acidification [19], regulates phagosome maturation [20,21] and NOX2 activity [22,23]. Functional cross-presentation is associated with limited proteolysis and reduced endocytic acidification, and NOX2 may play a central role by regulating phagosomal pH or the activities of proteolytic enzymes [24–27].

Here, we studied the effect of maturation on cross-presentation of particulate antigens, using HSV-1 infected cells as an antigen source as well as bead-bound OVA. We found that besides TLR ligands, cytokines like TNF-α are also able to negatively regulate cross-presentation of particulate antigens in mature DCs, whereas CD40L had no effect on cross-presentation of HSV-1 antigens. Phago-lysosomal acidification was increased in CpG or TNF-α matured DCs but most strongly in LPS matured DCs, and LPS maturation also had the strongest inhibitory effect on OVA cross-presentation, both for cells pretreated with LPS as well as when the DCs were exposed to LPS and antigen simultaneously.

## Material and Methods

### Mice

C57BL/6 (B6) and BALB/c were obtained from Jackson Laboratory (Bar Harbor, ME). Animals were housed and used according to Yale’s institutional guidelines. All animal work was conducted according to relevant national and international guidelines. Yale’s Institutional Animal Care and Use Committee approved the use of mice in this study. All cell lines described were of mouse origin and have been previously published.

### Cells

Bone marrow-derived DCs were prepared from mice between 6-12 weeks of age and cultured for 5-7 days with 1-2 medium replenishments without disturbing the cells. DCs were kept in RPMI 1640 (GIBCO, Life Technologies, Rockville, MD) with 10% FCS (Thermo, Fisher Scientific, Waltham, MA), 50 µM β-mercapthoethanol (Sigma) and 2mM L-glutamine, 100U/ml penicillin, 100mg/ml streptomycin (Pen/Strep), 12mM HEPES, non-essential amino acids (all GIBCO) and 20 ng/ml GM-CSF (R&D Systems). The HSV-1 gB_498-505_-specific MHC class I restricted hybridoma HSV-2.3.2E2 was a generous gift from G. Belz (Walter and Eliza Hall Institute of Medical Research) and was cultured as described [28,29]. The HSV gD_290-302_-specific MHC class II restricted hybridoma F1 was provided by A. Brooks (University of Melbourne) and cultured as for HSV-2.3.2E2. The RR1/ICP6_822-829_ – specific H-2Kb-restricted cytotoxic T-lymphocyte (CTL) clone 1D11 and corresponding stimulator cell line B6/T350 RR1_822-829_ were provided by R. Bonneau (Pennsylvania State University) and cultured as described [30]. The B3Z CD8^+^ T-cell hybridoma specific for OVA_257-264_ was a gift from N. Shastri (University of California, Berkeley) and grown in RPMI 1640 medium with 10% FBS, 2mM L-glutamine, Pen/Strep, 50µM 2-ME, 1mM pyruvate. HeLa and Vero cells were grown in DMEM with 10% FCS, L-glutamine and 20 µg/ml gentamicin.

### Maturation of DCs

DC maturation was induced on day 5 or day 6 of culture for 22h. Stimuli were added to the medium in 24 well plates without disturbing DC clusters. Immature control cells were left untreated. Stimuli used were 0.1 µg/ml LPS (*E. coli* 0111:B4, InvivoGen, San Diego, CA), 1µM CpG (ODN1668 5’-tccatgacgttcctgatgct-3’), 1 µg/ml recombinant mouse CD40L (PeproTech, Rocky Hill, NJ) and TNF-α (PeproTech) at 10ng/ml for gBT experiments, at 20ng/ml for B3Z experiments.

### Preparation of infected cells

HeLa cells were infected with HSV-1 (F strain) at a MOI of 5:1 for 1h in culture medium. Cells were washed and kept in medium containing 0.2mM Acyclovir (Calbiochem) for 10h. Residual virus was UV inactivated with 200mJ/cm^2^ in a Stratalinker 1800. HeLa-HSV were cultured for additional 4-6h before use as apoptotic bodies. To produce necrotic bodies, cells were subjected to three rounds of freeze/thaw after residual virus inactivation. Necrotic or apoptotic HeLa cells were re-suspended to 2.5x10^6^/ml in DC medium. Plaque assays with both cell preparations confirmed that no infectious virus could be recovered from either. For direct infection of DCs, HSV was added at an MOI of 3 to DCs in medium containing 10µg/ml gentamicin instead of Pen/Strep. After 1h of infection, DCs were washed three times and kept in acyclovir-containing DC medium for 12h before addition of T cells as described below. Vero cells were infected with recombinant OVA-expressing vaccinia virus (VV-OVA) at a MOI of 5 and chased for 7h before virus inactivation by UV irradiation. As a control for complete inactivation, VV-OVA-infected Vero were added to H2K^b^-expressing 293T cells instead of DCs, which did not activate the B3Z hybridoma (data not shown).

### Antigen presentation assays

For HSV cross-presentation assays, matured or untreated DCs were washed and fed with apoptotic or necrotic infected HeLa cells at a 4:1 (DC:HeLa) ratio for 5h in 96 well round bottom plates. Subsequently, 1x10^5^ gB- or gD-specific hybridoma T cells were co-cultured with 1x10^4^ antigen-loaded DCs for 20h. For co-culture with the RR1/ICP6 specific CTL, Vero cells were used instead of HeLa and 5x10^4^ DCs were co-cultured with 1x10^5^ CTL for 20h. For OVA bead cross-presentation, OVA was non-covalently bound to 3µm carboxylated or plain (similar results) latex beads (Polysciences). Immature or mature DCs were harvested from 24 wells, washed, seeded at 1.3x10^5^ DC/96 flat bottom well and pulsed for 5h with OVA-beads at 10:1 (bead:DC). In some cases, 1µM CpG or 0.1µg/ml LPS was added during the bead pulse, or immature DCs were pre-treated for 15min with 0.2µM epoxomicin that was maintained throughout the bead pulse. DCs were then washed and fixed in 1% PFA for 10min. Fixation was stopped with 200mM glycine in PBS, pH 7.4. After 2 washes, DCs were co-cultured with 1x10^5^ B3Z for 18h. For VV-OVA, DCs were fed apoptotic VV-OVA-infected Vero cells (DC:Vero ratio 4:1) for 5h, washed and fixed as above. 1x10^5^ B3Z cells were added to 0.5x10^5^ DCs for 18h. For all assays, IL-2 in co-culture supernatants was measured by ELISA according to the manufacturer’s protocol (BD Biosciences).

### Cytokine profile

DCs were treated in the same manner as for antigen presentation assays to mimic cytokine levels in T-dC co-cultures. After 22h of maturation, DCs were washed extensively and plated in 96 well plates, either with or without the addition of HSV-infected HeLa cells. After 24h, supernatants were harvested and IL-10, IL-12(p70), IFN-γ and TNF-α were measured with the Bio-Plex Pro^TM^ Mouse Cytokine Assay on a Bio-Plex System (Bio-Rad) according to the manufacturer’s instructions. Alternatively, supernatants of matured DC co-cultures with infected HeLa cells were used for transfer experiments and added to T-dC co-cultures during antigen presentation assays with untreated DCs.

### Flow cytometry

The following antibodies and appropriate isotype controls (all BD Biosciences) were used: anti-H-2K^b^ (AF6-88.5), anti-I-A/I-E (M5/114.15.2), anti-CD40 (3/23), anti-CD86 (GL1), anti-CD70 (FR70), PD-L1 (MIH5), PDL-2 (TY25), all PE conjugated and APC conjugated anti-CD11c (HL3). Rb anti-ovalbumin (Polysciences), and A647-conjugated goat anti-mouse F(ab’)_2_, A488-conjugated goat anti-rabbit antibody F(ab’)_2_ (highly cross-adsorbed) and A647-conjugated goat anti-rabbit F(ab’) (highly cross-adsorbed) Invitrogen/Molecular Probes). Data was collected on a FACSCalibur (BD Biosciences) and analyzed with FlowJo software (Tree Star).

### Phagocytosis assays

Untreated or matured DCs were allowed to phagocytose HSV-infected necrotic HeLa bodies, labeled with the fluorescent membrane binding dye PKH26 (Sigma), for 4h at a 4:1 (DC:HeLa) ratio in 200µl complete DC medium, mimicking conditions of antigen presentation assays. One sample with immature DCs was kept on ice during the 4h uptake. Another sample was pre-treated for 20min with 2.5µg/ml cytochalasin D (Cyt D) and the drug was maintained during the entire uptake incubation at 37°C. After 4h, all samples were washed 2x in cold PBS-1% FBS, labeled on ice with anti-CD11c APC and fixed in 2% PFA after 2 additional washes. For bead uptake, 2.5x10^6^ DC/ml were incubated with OVA coated carboxylated YG beads at a 3:1 ratio for 10min or 1h at 37°C. Samples were washed over 2ml cold FBS at 150g, Fc-receptors blocked (Mouse BD Fc Block), extracellular beads stained with rabbit anti-OVA antibody followed by A647-conjugated goat anti-rabbit F(ab’)_2_, and finally fixed in 2% PFA-PBS. As controls, LPS matured DCs or immature DCs were kept on ice or treated with CytD as described above. Phagocytosis was assessed by flow cytometry by gating on DCs.

### Phagosomal acidification

1x10^6^ immature or mature DCs were re-suspended in 50µl CO_2_-independent medium (Invitrogen). A vial of pHrodo labeled *E. coli* (Invitrogen) was re-suspended in CO_2_-independent medium, sonicated for 5min and 220µl were added to each DC sample. After 30min pulse in a 37°C water bath, samples were washed over 2ml FBS at 150g. Fresh DC medium with 20mM HEPES was added and DCs were chased for 5 min (t1), 1.5 h (t2) and 3.5h (t3). After each time-point, emission of pHrodo fluorescence was acquired immediately by flow cytometry. Since there were differences in uptake between the samples, fold increase of pHrodo fluorescence over time was determined for each sample (MFI FL-2 (t3)/ MFI FL-2 (t1)) and compared to the increase of pHrodo fluorescence occurring in immature DCs. To compare different experiments, the fold increase of pHrodo fluorescence over time in immature DCs was set to arbitrary units 1.0 and the fold increase of fluorescence in mature samples expressed relative to this number. To ensure that emission of fluorescence was coupled to intracellular acidification, immature and mature DCs incubated for 3h with pHrodo labeled *E. coli* were fixed, permeabilized for 10min in 0.1% Triton X-100 and protease inhibitor cocktail (Roche) containing potassium hydrogen phosphate buffer pH 5.8 on ice. Cells were then kept for 50min in acidic buffer without detergent and assessed by flow cytometry, gating on DCs containing *E. coli*. In parallel emission of pHrodo-E.coli alone in either acidic buffer or at pH7.4 was recorded.

### GILT Western Blots

Immature and matured DCs were sorted with CD11c magnetic beads (Miltenyi Biotec), lysed in PBS containing 1% Triton X-100 and protease inhibitors (Roche) for 1h on ice. Soluble material was separated by non-reducing SDS-PAGE and GILT was detected by western blot using rabbit anti-mouse GILT serum and Grp94 by rat anti-Grp94 (Enzo Life Sciences). HRP-conjugated secondary reagents (Jackson ImmunoResearch) were used and blots developed with ECL plus (GE Healthcare). For quantification purposes, blots were scanned on a fluorimager and analysed with ImageQuant software (Molecular Dynamics) software.

### OVA degradation assays

Phagosomal degradation of bead-bound OVA was carried out essentially as described in detail in reference [31]. The general outline of the process and modifications are described here. Immature or mature DCs were pulsed for 5min with beads coated with a combination of OVA andBSA, (bead:DC ratio 3:1) stopped with cold PBS, washed 2x over cold FBS (5min, 150g) and either processed directly (chase t=0) or chased for 1.5h. For certain immature or matured samples, CpG or LPS were added in combination with OVA/BSA beads. One sample of immature DCs was pre-treated with 25nM concanamycin B (ConB) for 20min and bead incubation was performed in the presence of the drug. For all samples (t=0 and t=1.5h), extracellular beads were marked with mouse anti-BSA antibody (7G10, Abcam) followed by A647-conjugated F(ab’)_2_ of goat anti-mouse IgG before cell lysis. Cells were lysed in 50mM Tris pH 7.4, 0.5% NP40, Roche protease inhibitors, 200µg/ml DNAse for 1h on ice. Lysates were centrifuged at 200g for 5min to pellet cell debris prior to bead recovery from supernatants. Free beads were stained for intact and degraded OVA with rabbit anti-OVA antiserum followed by A488-conjugated goat anti-rabbit IgG antibody. Mean fluorescence intensities of OVA were detected by flow cytometry, gating on single beads. Extracellular beads were excluded based on staining by the anti mouse IgG reagent.

### Statistical analysis

Statistical analysis of ELISA, FACS and cytokine bead-assay data was performed using one-way ANOVA with Bonferroni’s post-hoc test for comparison of multiple conditions. For comparison of two data sets, Student’s two-tailed t-test was applied, indicated in figure legends.

## Results

### Matured DCs have decreased capacity for cross-presentation

**Figure 1:**
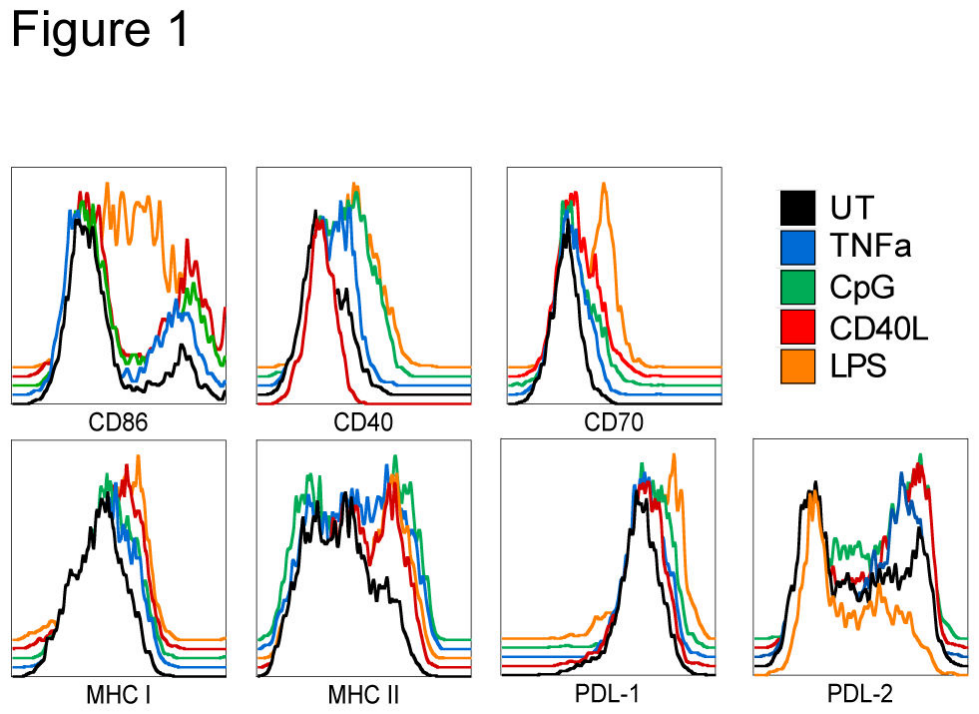
Phenotype of matured DCs. DCs matured as described in the methods section were labeled with anti-CD11c and either anti-CD86, anti-H2k^b^ (MHC I), anti-I-A/I-E (MHC II), anti-CD40, anti-CD70, anti-PDL-1 or anti-PDL2 and analyzed by flow cytometry gating on CD11c+ cells. Black histograms represent untreated DCs, DCs treated with 10ng/ml TNF-α are in blue, 1µM CpG in green, 1µg/ml CD40L in red and 0.1 µg/ml LPS in orange. Note that CD40L stimulated cells do not express surface CD40, likely due to receptor internalization.

**Figure 2:**
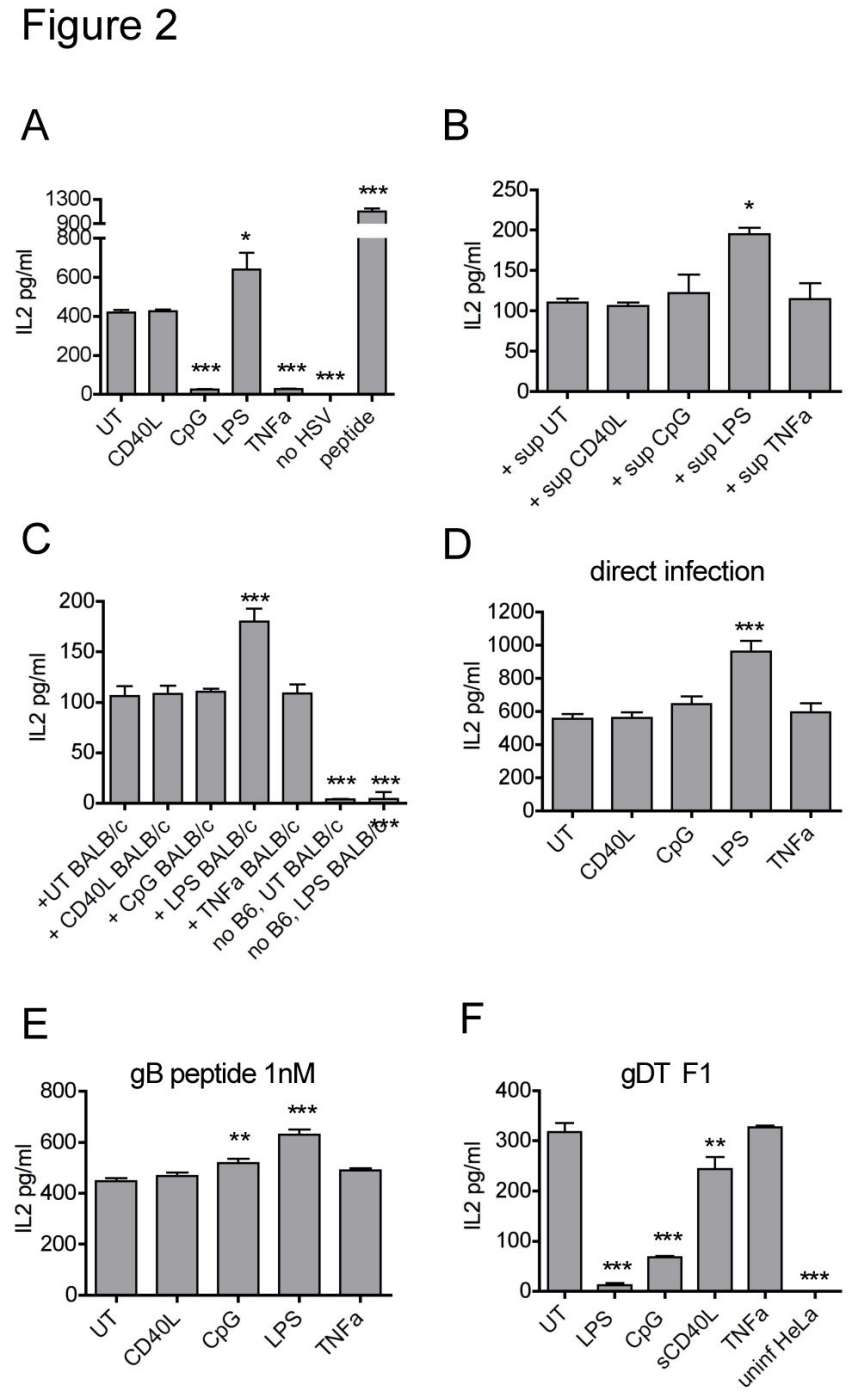
Antigen presentation of HSV-gB is inhibited in certain mature DCs. (A) DCs were activated for 22h with either 1µg/ml CD40L, 1µM CpG, 0.1µg/ml LPS, 10ng/ml TNF-α or left untreated, washed, then fed apoptotic HSV-1-infected HeLa cells for 5h and finally co-cultured with the MHC class I-restricted gB-specific hybridoma (gBT). For the peptide control, immature DCs were pulsed with 5nM gB_498-505_ peptide for 1h, washed and then co-cultured with the hybridoma T cells. IL2 was measured by ELISA after 20h. (B) Immature DCs were fed HeLa-HSV and subsequently co-cultured with gBT in the presence of supernatant collected from either immature DCs (“+sup UT”) or supernatants of matured DCs. The supernatants were prepared in a manner mimicking cytokine levels in gBT-DC co-cultures: immature DCs were matured for 22h with the various maturation stimuli or left untreated, washed extensively and fed HSV-infected HeLa in 96 well plates. After 24h, supernatants were removed. (C) Immature DCs were fed HeLa-HSV and co-cultured with gBT in the presence of immature (UT) BALB/c DC or BALB/c DCs matured with either 1µg/ml CD40L, 1µM CpG, 0.1µg/ml LPS or 10ng/ml TNF-α. (D) Immature (UT) or mature DCs were directly infected with HSV and co-cultured with gBT. (E) Immature (UT) or mature DCs were pulsed with 1nM gB peptide for 1h, washed and co-cultured with gBT. (F) Immature (UT) or mature DCs were stimulated and fed with HeLa-HSV as in (A) and then co-cultured with the MHC II –restricted gD-specific T cell hybridoma gDT F1. (A) -(F) IL-2 measured by ELISA after 20h. One representative of three experiments is shown for (A) and (F), one of two for (B)-(E). Error bars indicate SD of biological triplicates, * p<0.05, ** p<0.01, *** p<0.001.

LPS, CpG, TNF-α and CD40L were chosen as representative stimuli to induce DC maturation and used at concentrations that caused at least a degree of phenotypic maturation and/or had an effect on cross-presentation (Figure 1). To address whether mature DCs are capable of presenting newly encountered antigens from virally infected cells, immature untreated (UT) or matured DCs were fed apoptotic HSV-infected HeLa cells and then used to stimulate an HSV-glycoprotein B (gB)-specific T cell hybridoma. Similar results were obtained using freeze-thawed HSV infected HeLa cells (data not shown). Cross-presentation was impaired in DCs matured with 1µM CpG or 10ng/ml TNF-α but not by 1µg/ml CD40L (Figure 2A). LPS at 0.1µg/ml increased cross-presentation, however we have previously reported that the increase of gB cross-presentation by LPS is at least to some extent due to co-stimulation [18]. To exclude that the observed effects by TNF-α and CpG resulted from changes other than in cross-presentation and to confirm that co-stimulation contributes to the effect of LPS, a series of control experiments was performed. Cytokines (IFN-γ, TNF-α, IL-10 and IL-12) produced by immature or mature DCs after co-culture with HSV-infected cells were measured under conditions mimicking the situation in T cell co-cultures (Figure S1). Although immature DCs induced more TNF-α than CpG- or LPS-matured DCs, overall cytokine levels were low (Figure S1). Only IL-10 was up-regulated in all samples after DC culture with HSV-infected cells, with CpG, TNF-α and CD40L matured DCs producing similar levels of IL-10 as UT DCs and LPS-DCs producing less (Figure S1). To rule out that other cytokines or the cytokine combination secreted by the differently matured DCs interfered with cross-presentation, supernatants from immature (“sup UT”) or matured DCs incubated with HSV-infected cells were added to co-cultures of gB T cells with untreated, HSV-1/HeLa-loaded DCs during antigen presentation assays (Figure 2B). Apart from the co-stimulatory effect of LPS-DC supernatant, the influence of soluble mediators from other matured DCs was negligible for the outcome of cross-presentation. In addition, mature BALB/c DCs, which could deliver co-stimulatory signals in trans but not present the gB peptide to the H2K^b^-restricted hybridoma, were able to enhance cross-presentation when stimulated with LPS, washed and added to gBT/B6DC/HeLaHSV co-cultures, but were unable to abrogate cross-presentation when stimulated with CD40L, CpG or TNF-α (Figure 2C). Finally, neither mature DCs that were directly infected with HSV nor mature DCs pulsed with the gB peptide were impaired in their ability to stimulate the T cell hybridoma (Figure 2D, E). In parallel, we assessed the ability of the matured DCs to present HSV antigens on MHC class II (MHC II) using the glycoprotein D (gD)-specific hybridoma (Figure 2F). As expected, TLR ligands CpG and LPS abrogated MHC class II presentation. CD40L decreased MHC II presentation, but in contrast to its effect on cross-presentation, 10ng/ml TNF-α did not impair MHC II presentation, underscoring that these two processes are regulated differently.

**Figure 3:**
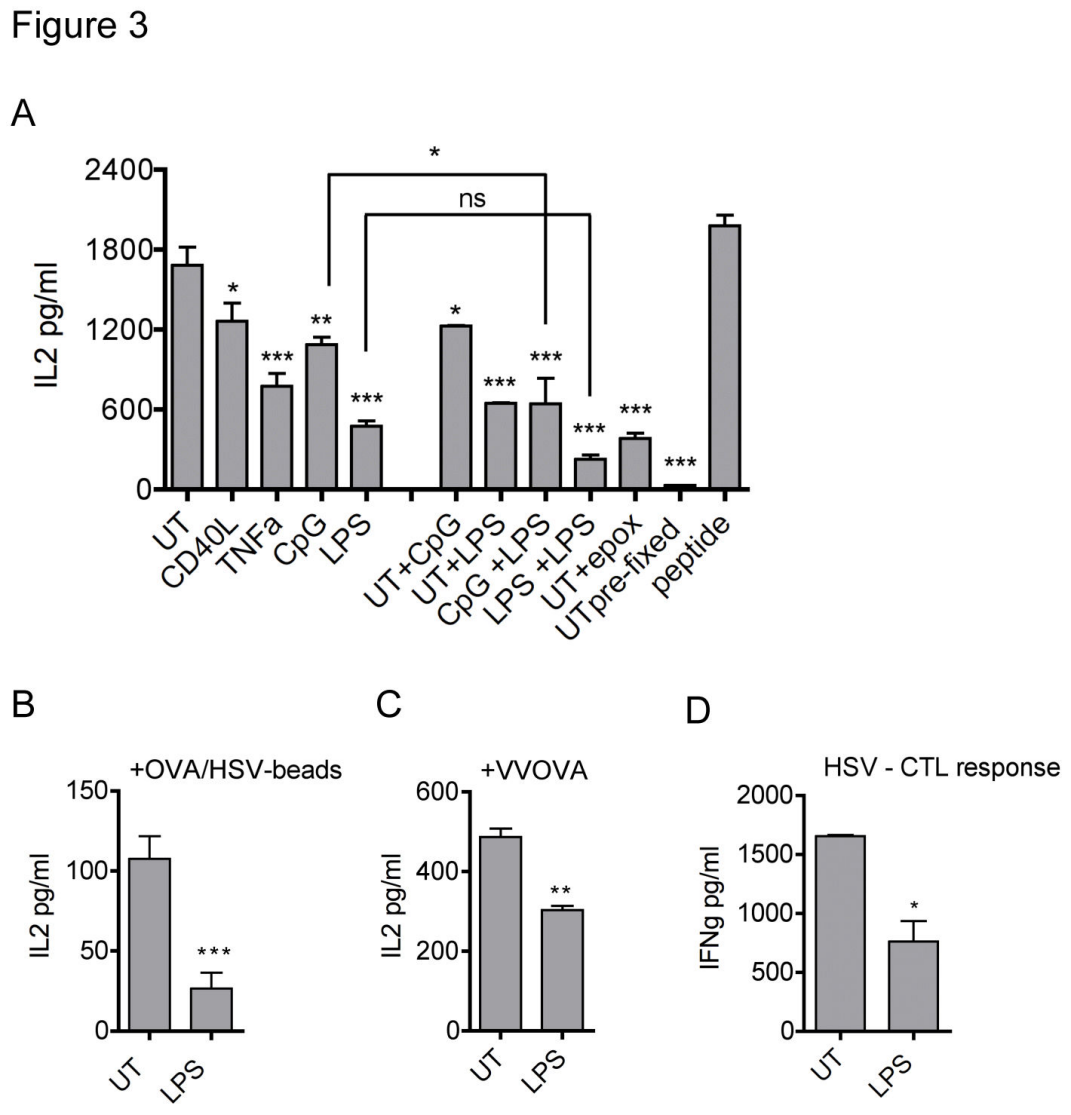
OVA cross-presentation is inhibited in most matured DCs. (A) Immature DCs were activated for 22h with 1µg/ml CD40L, 1µM CpG, 0.1µg/ml LPS or 20ng/ml TNF-α or left untreated (UT). Then, OVA-coated beads were added for 5h. For samples indicated with “+”, UT or CpG or LPS matured DCs were incubated with beads in combination with additional CpG, LPS or epoxomicin. As controls, one sample of UT DCs was pulsed with 1nM SIINFEKL peptide for 1h instead of beads, another UT sample was fixed before bead addition. After bead uptake, DCs were washed and fixed in 1% PFA for 10min. Fixation was stopped with 200mM glycine in PBS, pH 7.4. After 2 washes, DCs were co-cultured with B3Z for 18h, IL-2 measured by ELISA. One experiment of three is shown, error bars depict SD of biological triplicates. For statistical analysis, all samples were compared to UT DCs (first column) and additionally, CpG and LPS matured DCS were compared to the respective matured samples incubated with extra LPS during bead uptake, one-way ANOVA with Bonferroni’s post-hoc test, * p<0.05, ** p<0.01, *** p<0.001. (B) DCs were activated, incubated with OVA-beads and co-cultured with B3Z as described in (A) except for that latex beads had been coated with OVA in the presence of necrotic HeLa-HSV. One experiment of two is shown, error bars depict SD of biological triplicate samples, Student’s t-test *** p<0.001. (C) DCs were activated and co-cultured with B3Z as described in (A) with the modification that instead of OVA-beads, dead UV-irradiated Vero cells infected with recombinant OVA expressing vaccinia-virus were used. One experiment of two is shown. Error bars depict SD, Student’s t-test ** p<0.01. (D) LPS maturation also decreases cross-presentation of the HSV antigen ICP6. DCs were activated for 22h with 0.1µg/ml LPS or left untreated, then fed apoptotic HSV-infected Vero cells as described in the methods section DCs were subsequently co-cultured with an MHC class I-restricted RR1/ICP6-specific T cell line. IL2 was measured by ELISA, error bars indicate SD of biological triplicates, Student’s t-test * p<0.05. One experiment of two is shown.

OVA is the predominant antigen used to study cross-presentation, so we similarly investigated the effects of the different maturation stimuli on the cross-presentation of OVA non-covalently bound to latex beads. Again, CpG and TNF-α down-regulated cross-presentation, although the blocking effect was not as complete as in the gB system (Figure 3A). CD40L also diminished OVA cross-presentation to some extent. Virally infected cells contain a mix of TLR- and other stimulating ligands that may modulate cross-presentation in synergy with the maturation stimuli that were applied prior to phagocytosis. To test the effect of TLR ligands encountered at the time of phagocytosis, LPS or CpG were given simultaneously with the OVA-beads, either to immature (UT) DCs or to matured DCs (Figure 3A, “+LPS”, “+CpG”). DCs given a combination of TLR ligand and OVA beads cross-presented significantly less well than the same DCs (UT or CpG matured) incubated with OVA beads alone; for LPS matured DCs, the decrease in cross-presentation after extra addition of LPS during bead incubation did not reach statistical significance (Figure 3A).

LPS was the most potent inhibitor of OVA cross-presentation (Figure 3). As the increase of HSV-gB cross-presentation by LPS was at least partly a result of co-stimulation (Figure 2), and isolated other effects of LPS on gB cross-presentation could thus not be assessed, we wanted to address if LPS would still reduce OVA cross-presentation in the context of viral infection. LPS matured DCs also showed reduced cross-presentation when necrotic HSV-infected cell lysates were coated together with OVA onto latex beads (Figure 3B) or when OVA was delivered by cells infected with a recombinant vaccinia virus (Figure 3C). LPS matured DCs were also impaired in presenting the HSV-protein RR1/ICP6 after phagocytosis of HSV-1 infected cells to the respective cytotoxic T cell clone (Figure 3D). Together, these results suggest that LPS matured DCs, like CpG or TNF-α treated cells, generally cross-present less well than immature DCs and that the increase in response seen with HSV-gB is specific for either the gB antigen or the T cell hybridoma.

### Decreased uptake is not the major reason for inhibition of cross-presentation

**Figure 4:**
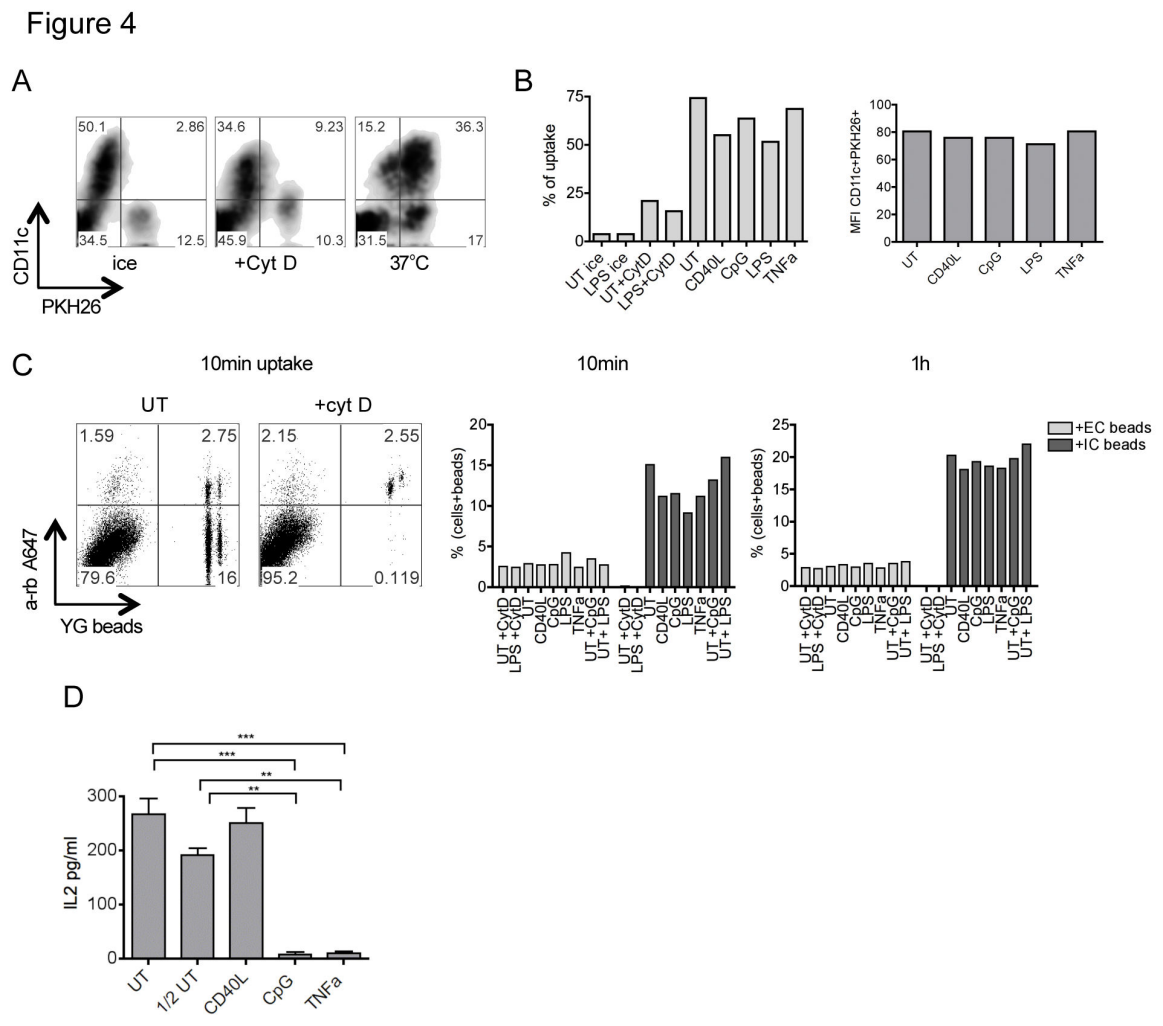
Differences in uptake cannot explain block in cross-presentation of mature DCs. (A) Representative uptake of apoptotic infected PKH26 labeled HeLa by DCs. As control, uptake was performed on ice or at 37°C in presence of Cytochalasin D (Cyt D). (B) uptake in CD11c+ populations gated as in (A) is shown for immature and mature DCs. Graphs show % of DCs (left panel) and mean fluorescence intensity (MFI; right panel) of DCs that have internalized infected cells. One representative experiment of 3. (C) Uptake of OVA-coated YG beads by mature and immature DCs, or immature DCs fed with beads in combination with CpG or LPS as indicated by “+”. As control, uptake for immature or LPS matured DCs was performed in presence of CytD. Extracellular beads on DCs were stained by anti-OVA followed by a-rb A647. In the presence of Cyt D, only DCs with extracellular beads are detected (left). The % of cells with either extracellular (EC) or intracellular (IC) beads after a 10min uptake (middle) or 1h uptake (right) is shown. (D) immature (UT) and DCs matured with indicated stimuli were fed apoptotic HSV-1-infected HeLa cells for 5h. One of the untreated DC samples was fed only ½ the amount of HSV-HeLa (label “½ UT”). DCs were then co-cultured with the MHC class I-restricted gB-specific hybridoma (gBT). IL2 was measured by ELISA after 20h. Error bars depict SD of biological triplicates, ** p<0.01, *** p<0.001.

To test whether reduced phagocytosis could explain the decrease in cross-presentation of matured DCs, we compared the ability of immature and mature DCs to take up necrotic HSV-infected HeLa cells that were labeled with the membrane dye PKH26 (Figure 4A, B). Incubation on ice or addition of cytochalasin D prevented uptake, indicating that the assay reflected genuine phagocytosis (Figure 4A). Although showing some decrease in uptake compared to untreated DCs, DCs treated with each of the maturation stimuli still took up considerable amounts of labeled material (Figure 4B).

A similar result was obtained in phagocytosis assays using OVA coated yellow-green dyed beads (Figure 4C), where it was possible to exclude DCs bearing adherent extracellular beads by anti-OVA staining (Figure 4C, left panels). The differences between immature and mature DCs that had internalized beads were more pronounced at early times (Figure 4C, middle panel). After 10min of incubation, approximately 30% of LPS treated, bead-positive DCs had adherent extracellular beads, compared to only about 15% in untreated DCs. Also, LPS treated DCs usually showed a 25-35% decrease in uptake compared to immature DCs in the first 5-20 min and only about 10-15% decrease after 1h or more (Figure 4C, right panel), most likely because extracellular adherent material is internalized over time. Similar results were obtained when the percentage uptake was compared after gating on distinct DC populations that had internalized 1 bead or two beads (the number of cells having internalized 3 or more beads was too small to draw any conclusion) (Figure S2).

The fact that matured DCs still phagocytose antigens reasonably well, together with the observation that CD40L matured DCs displayed a similar decrease in uptake to the other matured samples while cross-presentation was not impaired to the same extent, suggests that impaired phagocytosis alone cannot explain the defect in cross-presentation. In particular not in the HSV-gB system, where CpG and TNF-α matured DCs hardly cross-presented at all (Figure 2A). To further clarify this issue, immature DCs were incubated with half the amount of HeLa-HSV than matured DCs, which resulted in comparable antigen uptake (Figure S3). In a subsequent cross-presentation assay, untreated DCs loaded with half the antigen stimulated gBT cells somewhat less well than those with a full antigen load (difference not reaching statistical significance, Figure 4D). However, this reduced response was significantly higher than the responses generated by CpG or TNF-α matured DCs (Figure 4D), arguing that reduced uptake is not the determining factor regulating cross-presentation.

### Phagosomal acidification progresses faster in LPS matured DCs

**Figure 5:**
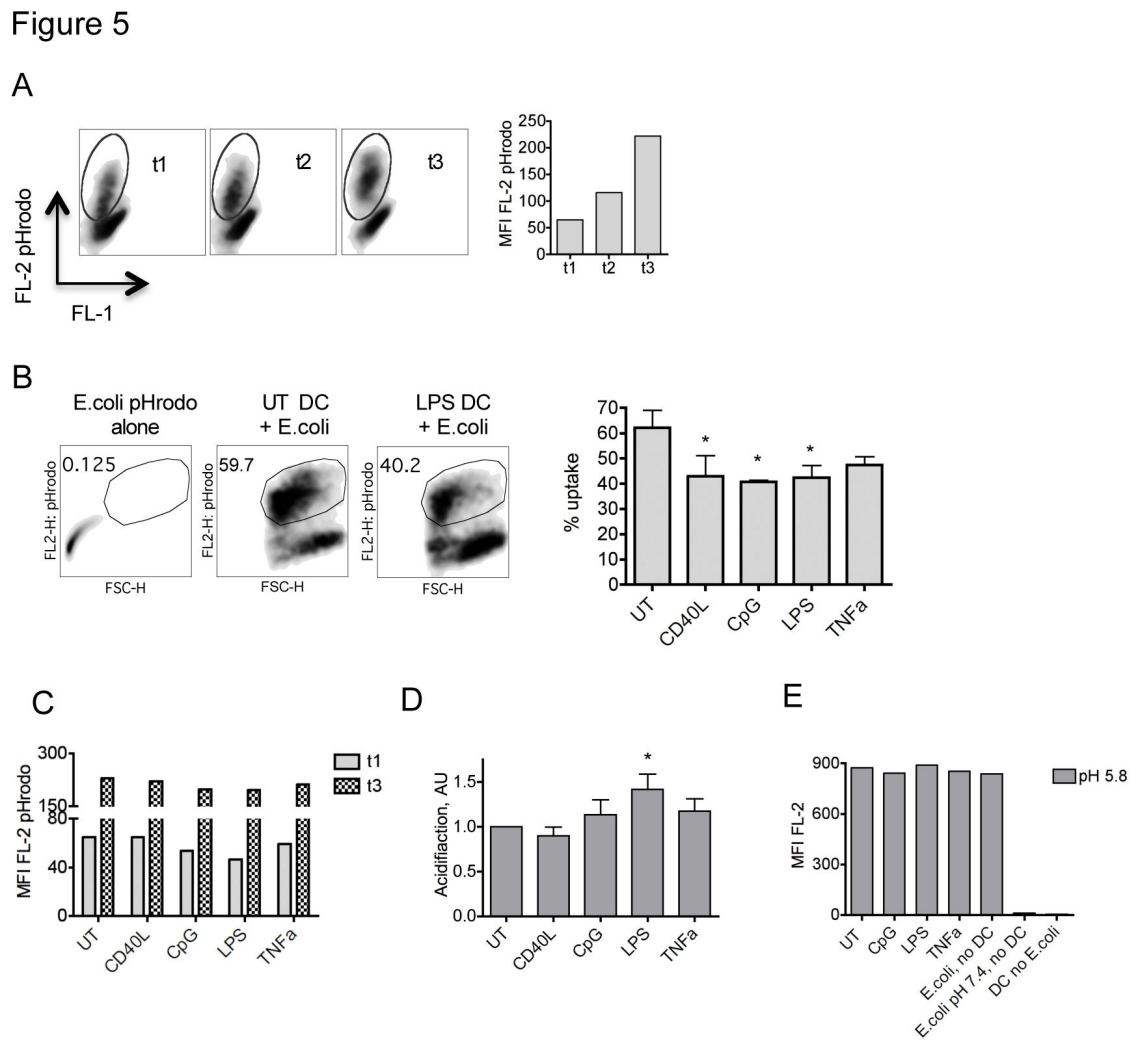
Rate of phagosomal acidification in matured and immature DCs. Immature (UT) and mature DCs were pulsed with pHrodo labeled *E. coli* for 30min, washed and assessed for emission of red fluorescence after additional 5min (t1), 1.5h (t2) and 3.5h (t3) of chase. (A) Representative example for the increase in pHrodo fluorescence by DCs with internalized *E. coli* over time. (B) Immature (UT) and matured DCs showed differences in uptake of pHrodo-E.coli. One representative gating example displaying the percentage of UT or LPS matured DCs containing internalized *E. coli* after 3.5h is shown on the left. On the right, differences in uptake of 3 independent experiments are summarized, error bars indicate SD, * p<0.05. (C) Mean fluorescence intensity (MFI) of immature or matured DCs with internalized pHrodo-E.coli after 5min (t1) and 3.5h (t3) of chase is shown for one representative experiments out of three. (D) The fold increase of fluorescence over time (MFI FL-2 (t3)/ MFI FL-2 (t1)) was calculated for each matured DC sample and expressed relative to the fold increase of fluorescence obtained for immature (UT) DCs in the same experiment. Summary of three independent experiments, error bars depict SD, * p<0.05. (E) Immature (UT) and matured DCs incubated with pHrodo labeled *E. coli* were fixed, permeabilized and re-suspended in an acidic buffer, pH 5.8. As control, emission of pHrodo-E.coli alone in either acidic buffer or at pH7.4 is shown as well. One representative of 2 experiments is shown.

To compare the rate of phagosome acidification, immature and matured DCs were fed *E. coli* labeled with pHrodo, a dye that is non-fluorescent at neutral pH but emits red fluorescence in an acidic environment. We opted to use a flow cytometry based read-out, which allowed us to gate on DC populations that have taken up particles and follow the acidification rate over-time in the same sample (Figure 5A), eliminating confounding effects of differences in uptake (Figure 5B). Immature and mature DCs were pulsed with *E. coli* pHrodo, washed and assessed for emission of red fluorescence after 5min (t1), 1.5h (t2) and 3.5h (t3) of chase (Figure 5A and C). The fold increase of mean fluorescence intensity over time in DC populations with internalized *E. coli* pHrodo was used to measure the acidification rate (Figure 5C and D). To compare different assays, the increase in acidification for the various matured DCs was normalized to the value obtained for immature DCs in the same experiment (Figure 5D). CD40L treated DCs were similar to untreated DCs, while CpG, TNF-α and LPS matured DCs showed a trend towards increased acidification. However, only the difference between UT and LPS DCs reached statistical significance (Figure 5D). To ensure that emission of fluorescence was coupled to intracellular acidification, DCs chased for 3h after incubation with *E. coli* pHrodo were fixed, permeabilized and re-suspended in an acidic buffer, which resulted in the same fluorescence intensity for all samples as well as *E. coli* in acidic buffer alone (Figure 5E).

### The rate of intra-phagosomal OVA degradation is similar for matured and untreated DCs

**Figure 6:**
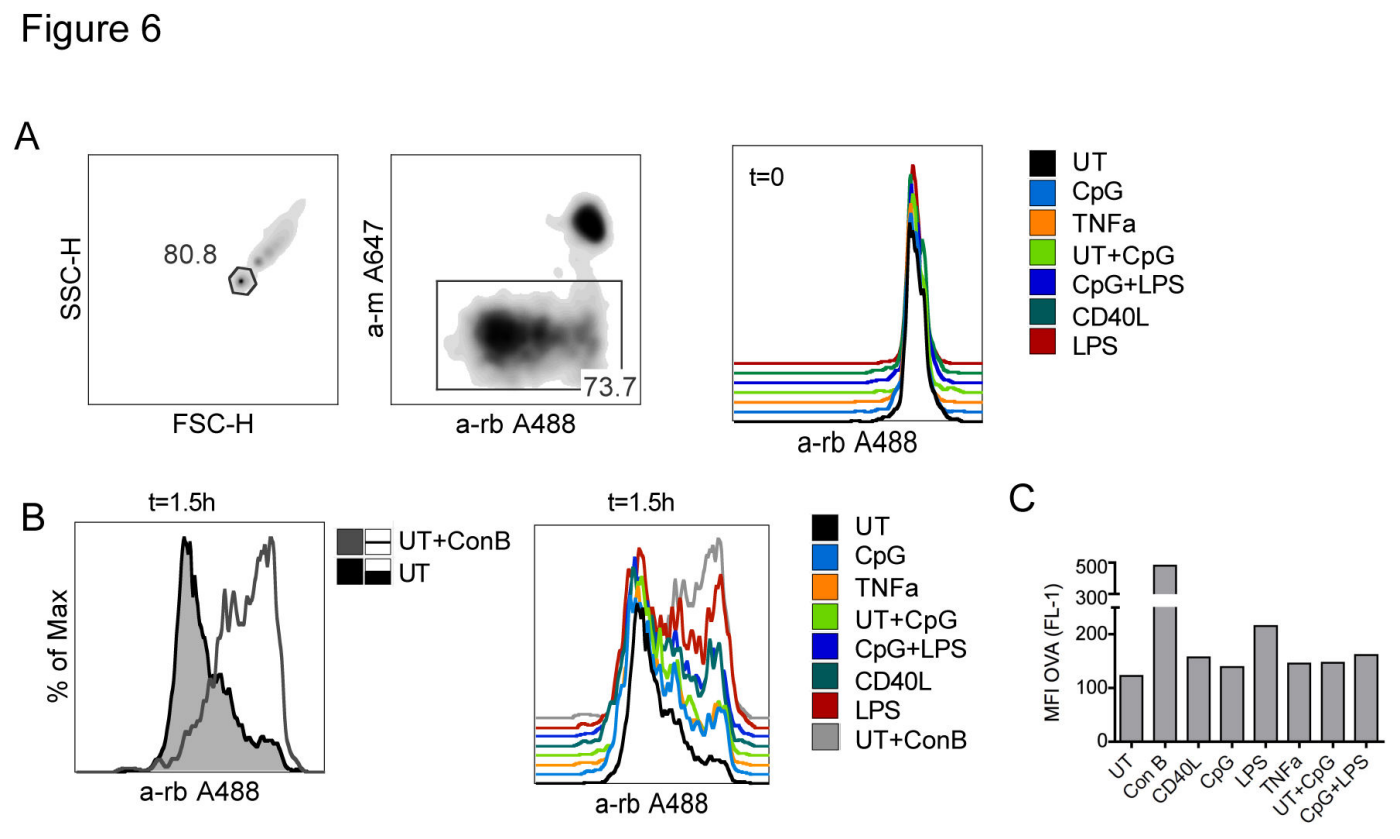
OVA degradation in phagosomes of matured and immature DCs. Immature or mature DCs were pulsed with OVA/BSA coated beads for 5min, washed and separated into two samples. One sample was chased for 1.5h and then stained for BSA while the other sample (t=0) was stained directly with m anti-BSA followed by anti-m A647 to mark non-internalized beads. After BSA staining, cells were lysed in 0.5% NP40. Beads recovered from lysates were stained for intact and degraded OVA with rb anti-OVA followed by anti-rb A488. For samples indicated with “+”, CpG or LPS was added in combination with OVA/BSA beads during pulse. (A) Intensities of OVA fluorescence were detected by flow cytometry, gating on single beads (left panel). Extracellular beads were excluded based on a-m A647 staining (middle panel). At t=0 (after pulse), all samples showed a uniform, non-degraded OVA peak (right panel). (B) After 1.5h chase, control samples chased in the presence of Concanamycin B had reduced OVA degradation (both panels). OVA degradation patterns for all samples are shown in the right panel, the respective MFI is graphed in (C). One representative of three is shown.

Because of the observed differences in phagosomal acidification between untreated and LPS treated DCs, we hypothesized that there would be a related difference in the rate of phagosomal degradation of the cross-presented antigen. To address this, we followed degradation of OVA on OVA/BSA coated beads recovered from phagosomes (Figure 6). Residual extracellular beads were marked with anti-BSA prior to cell lysis and excluded from analysis (Figure 6A, middle panel). After five minutes of uptake (t=0 of chase), all samples showed intact OVA protein on internalized beads (Figure 6A, right panel). After 1.5h of chase, immature control DCs that had phagocytosed beads in the presence of concanamycin B (ConB), a specific inhibitor of vacuolar-type H^+^-ATPase that blocks acidification, showed much less degradation compared to immature (UT) DCs without the drug (Figure 6B, left panel). However, there was no difference in OVA degradation between immature (UT) DCs, CD40L-, CpG-, or TNFα- matured DCs, UT DCs that were fed OVA/BSA beads in combination with CpG, or CpG matured DCs fed with beads in combination with LPS (Figure 6B, right panel and 6C). The only exception were LPS matured DCs that had consistently more intact OVA on the beads than the other samples, which likely does not reflect a true attenuation of degradation but rather is a result of delayed internalization of adherent beads that could not be removed by washing at t=0.

### GILT maturation pattern differs between matured and untreated DCs

**Figure 7:**
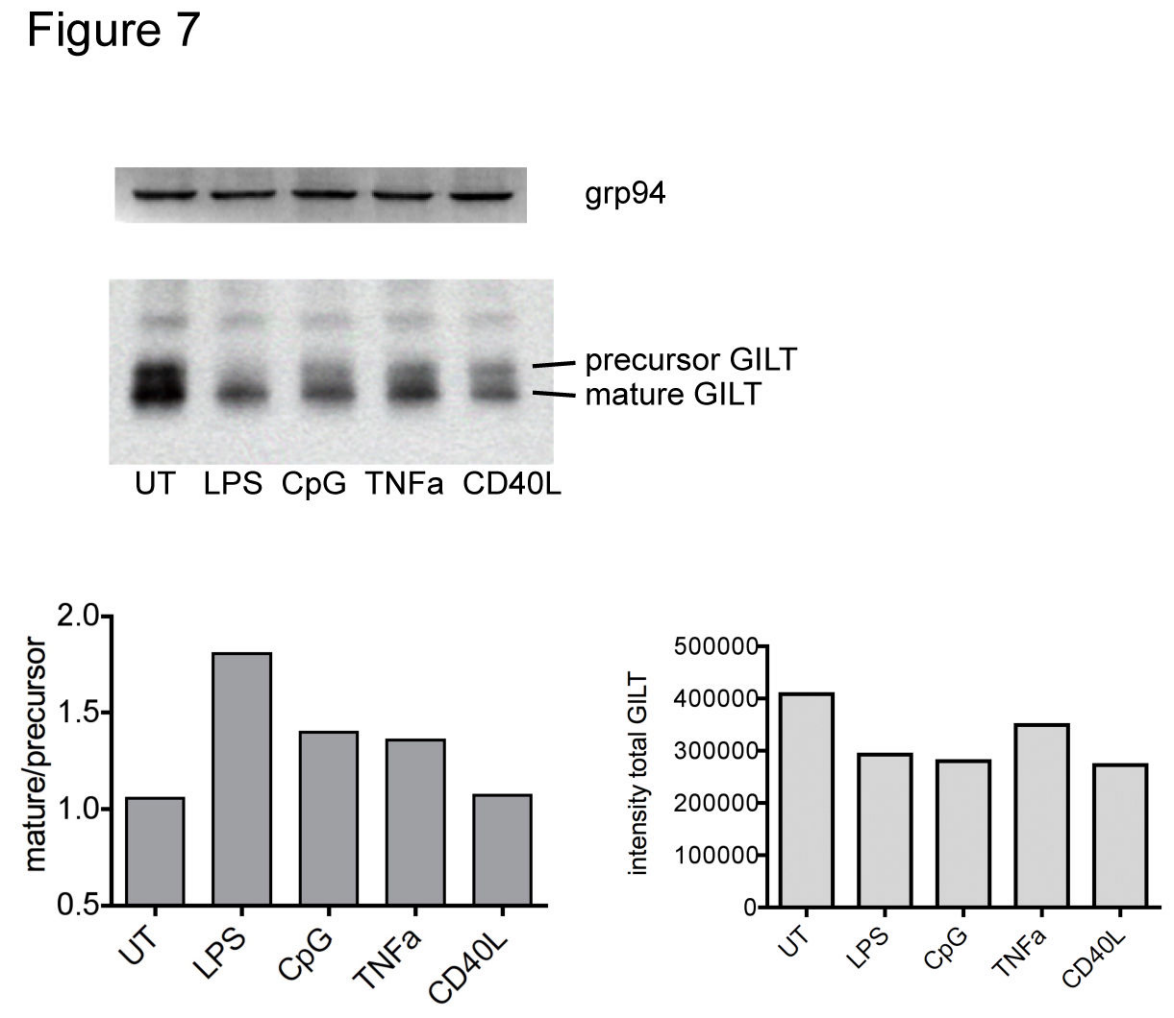
Lysosomal activation is higher in matured DCs. Immature (UT) or DCs matured for 22h with the indicated stimuli were sorted for CD11c, lysed and blotted for GILT with Tito (rb-anti GILT) followed by a-rb HRP (middle panel). Blotting for grp94 with rat anti-grp94 followed by a-rat HRP served as loading control (upper panel). Band intensities for precursor and mature form of GILT were quantified and the ratio obtained for each sample was plotted in the lower left panel, densitometry volume for total GILT (adjusted to grp94 loading control) is shown in the lower right panel.

Gamma-IFN inducible lysosomal thiol reductase (GILT) promotes both cross-presentation and MHC II presentation of antigens containing disulfide bonds [32–34]. The mature form of GILT is processed from a precursor by proteolytic cleavage at the N- and C-termini by other lysosomal proteases [35]. The ratio of proenzyme to mature form reflects lysosomal activation. To test whether immature and mature DCs differed in lysosomal activation, the ratio of precursor and mature GILT was assessed. Total GILT levels were higher in immature compared to matured DCs (Figure 7), perhaps, by analogy to human macrophages, due to secretion of GILT during maturation [36]. Untreated and CD40L treated DCs contained approximately equal amounts of mature and precursor GILT, CpG and TNF-α treated DCs had more mature than precursor GILT, and LPS treated DCs had the strongest shift towards the mature form (Figure 7). This pattern of differences was similar to the differences in cross-presentation efficiency of the samples, suggesting that endo-lysosomal fusion and enzyme processing may play a role in regulating cross-presentation.

## Discussion

Under which circumstances and how cross-presentation is regulated during DC maturation is a matter of ongoing research. Here, we demonstrate that pre-treatment of DCs with certain types of maturation stimuli, but not all, inhibits cross-presentation of newly acquired particulate antigens, either from HSV infected cells or from OVA coated latex beads.

Studies that found a decreased capacity of mature DCs to cross-present new antigens most commonly propose reduced antigen uptake as a mechanism [6,12,17]. We, and others [11,18], have demonstrated that cross-presentation can be inhibited in mature DCs by factors independent of uptake. In the present study, mature DCs were still capable of internalizing particulate antigens (dead infected cells, OVA beads or *E. coli*) and uptake was ruled out as determining factor for the outcome of cross-presentation, at least for HSV-gB. CD40L matured DCs phagocytosed similar amounts of HSV-infected material as DCs matured by other means but were not impaired in cross-presentation, while cross-presentation was almost abrogated in TNF-α or CpG matured DCs. In addition, immature DCs incubated with less antigen to adjust for differences in uptake still cross-presented much better than matured DCs. For cross-presentation of OVA however, decreased uptake may contribute to the outcome, since CD40L matured DCs that showed a decrease in uptake similar to TNF-α or CpG also had some reduction in cross-presentation, although less than seen for any of the other stimuli. Also as observed previously [18], the degree of phenotypic maturation that was induced by the various stimuli did not correlate with the outcome of cross-presentation.

Matured DCs were not generally refractory to signals by virally infected cells, as they further up-regulated co-stimulatory molecules (Figure S4) and were able to produce cytokines (Figure S1). Apart from co-stimulatory effects by LPS on gB antigen presentation, differences in cytokine production did not play a role for the outcome of cross-presentation. Of note, increased IL-10 production after contact with infected cells together with a changed ratio of other cytokines may well impact the outcome of cross-priming in vivo, which is not subject of investigation in this study.

An alternative explanation for changes in cross-presentation is the amount of antigen translocated to the cytosol. Reduced translocation could either be a consequence of altered assembly/recruitment of the (still ill-defined) translocation machinery or result from changes in phagosomal processing, altering the availability of suitable material for translocation. We did not see an obvious block in soluble OVA translocation during maturation (Figure S5). One caveat is that all published translocation assays are based on soluble proteins, such as quantification of cytosolic soluble OVA or enzymatic activity in the case of cytochrome c or HRP [6,37,38]. We cannot exclude that matured DCs were deficient in translocation of particulate antigens. Additionally, translocation rates may vary depending on the source of the antigen. For example, processing of HSV-gB from infected cells not only requires disassembly of ingested apoptotic bodies but also disulfide reduction by GILT [32], a process more complex than degradation of OVA bound to the surface of beads. It is conceivable that antigen-intrinsic factors were responsible for the bigger differences observed between mature and immature DCs in the cross-presentation of HSV-gB compared to bead-bound OVA.

Another level of complexity is the potential synergy of stimuli that are responsible for initial DC maturation and those acting simultaneously with acquisition of new antigens, such as during phagocytosis of virally infected cells. We demonstrated that such synergy does indeed exist, by combining LPS or CpG pre-treatment with administration of TLR ligand at the time of OVA-bead addition.

How TLR ligands in combination with phagocytic cargo affect phagosome maturation and intra-phagosomal antigen degradation is a matter of debate [20,21,39–41]. It has been proposed that only antigens that contain TLR ligands are routed to DC lysosomes in which efficient MHC II loading takes place [21]. Another study did not detect TLR-dependent modification of phagosome maturation in macrophages [41]. If TLR-dependent enhancement of phagosome maturation takes place in DCs, one could speculate that this increases antigen degradation that could negatively impact cross-presentation. On the other hand, it has been reported that phagosomes with beads internalized in the presence of LPS have a delayed acquisition of active proteases [42]. Here, we examined DCs post maturation to determine whether they have distinct degradation rates of cross-presented antigen. Despite having the most severe effect on down-regulation of OVA or ICP6 cross-presentation, in pilot experiments LPS matured DCs showed consistently much less OVA degradation than untreated DCs, when extracellular sticky beads were not excluded based on staining of a second antigen, BSA (data not shown). This result runs counter to the suggestion that inhibition of cross-presentation is coupled to enhanced degradation [19,24,25,42]. However, in our hands latex beads adhered strongly to LPS treated DCs, resulting in over-estimation of non-degraded OVA. Staining extracellular beads prior to cell lysis eliminated much background but could not completely prevent non-synchronized phagocytosis, precluding a definitive measurement of the OVA degradation rate in these cells. Adherence was less of a problem for CpG, TNF-α or CD40L treated DCs, and surprisingly, degradation rates of OVA were the same in matured and immature DCs, although OVA cross-presentation was differentially affected by maturation. Trombetta et al. found differences in intracellular antigen degradation between LPS matured and immature DCs for antigens more resistant to proteolysis, such HRP and HEL, but not for BSA [19].

If cross-presentation efficiency in matured DCs is influenced by permanent changes in phagosomal maturation, a critical parameter to consider is acidification. Plate-bound assays to follow phagosome maturation quantitatively were developed by Russell and colleagues [43,44] and are suitable for well-adherent macrophages. DCs are more difficult to handle, therefore we followed intra-phagosomal acidification by flow-cytometry, which also eliminated confounding effects from unequal antigen uptake of immature and matured DCs. Small increases in acidification were observed for TNF-α or CpG treated DCs compared to immature DCs that could possibly be meaningful and translate into significant functional changes, because activation of endolysosomal enzymes is tightly regulated by pH. However, despite mature DCs having increased acidification in late endosomal/lysosomal compartments, this may not be relevant to cross-presentation which is likely to occur in an earlier endosome or immature phagosome.

It is already known that LPS maturation enhances lysosomal proteolysis and acidification due to increased activity of the lysosomal vacuolar proton pump [19]. Consistent with this, LPS had the most dramatic effect on conversion of GILT from precursor to mature form in this study. GILT is constitutively expressed in professional antigen presenting cells and is essential for gB cross-presentation [32]. GILT, in both precursor and mature form, is enzymatically active even at neutral pH, although its optimum activity is at pH 4.0 to 5.0, similar to other lysosomal enzymes [35,45]. Therefore, GILT’s essential contribution to gB reduction during cross-presentation is not necessarily confined to late endosomal or lysosomal compartments. Early endosomal compartments with reduced proteolytic activity have been proposed as sites of antigen transfer to the cytosol for cross-presentation [38,46,47]. It is unlikely, however, that changes in GILT maturation state between immature and mature DCs have direct consequences on cross-presentation of gB. It is more plausible that enhanced acidification in the endosomal pathway has other consequences that impact cross-presentation, such as changing proteolysis rate for certain antigens or pH-dependent modifications of proteins involved in either translocation or intra-phagosomal peptide loading.

CD40L-induced maturation of DCs had little effect on cross-presentation, and had similar acidification rates in the endocytic system, reflected by similar GILT precursor/mature form ratio as immature DCs. CD40 is one potential candidate for targeting antigens to DCs during immunotherapy [48]. Together with other signaling pathways, CD40 can elicit effective immune responses. The fact that CD40L matured DCs retain most of their ability to cross-present could be beneficial, in terms of immunization for CD8-positive T cell responses, compared to other adjuvants which result in loss of this function.

## Supporting Information

Figure S1 **Cytokine secretion of immature and mature DCs with and without addition of HSV-infected HeLa cells.** The cytokine profile of untreated or matured DCs was assessed for the indicated analytes with a magnetic bead-based assay. DCs were matured for 22h or left untreated, washed, and re-cultured with our without HSV-1 infected HeLa cells for another 20h prior to supernatant collection. Supernatants from 3 independent experiments were used. Error bars depict SD, ** p<0.01, *** p<0.001.
(TIF)

Figure S2 **Uptake of OVA-coated YG beads.** Mature and immature DCs were fed OVA coated beads as described in the methods section. For samples indicated with “+”, beads were added in combination with CpG, LPS or cytochalasin D. DCs with extracellular beads attached were excluded based on anti-OVA staining followed by a-rb A647. The percentage of cells containing either 1 bead, 2 beads or 3 beads after a 10min uptake (upper panel) or 1h uptake (lower panel) is shown.
(TIF)

Figure S3 **Antigen uptake titration in immature and CpG matured DCs.** To identify a dose of antigen where immature DCs take up comparable amounts as matured DCs, DCs were prepared and pulsed with different amounts of CFSE-labeled necrotic HSV-infected HeLa cells in 96 wells as for antigen presentation assays. DCs were pulsed with usual amounts of HeLa-HSV (DC:HeLa ratio 4:1), half the amount (“1/2 Ag”) or one tenth (“1/10 Ag”). After 4h, uptake was assessed by flow cytometry. Percentage of uptake is calculated as CFSE+CD11c+ fraction of CD11c+ DCs.
(TIF)

Figure S4 **Matured DCs are not refractory to activating ligands in virally infected cells.** Matured DCs up-regulate co-stimulatory molecules further after encounter of virally infected cells. Immature (UT) or matured DCs were cultured as for cross-presentation assays with HSV-infected HeLa cells. Phenotype was assessed prior and post co-culture with infected HeLa cells, MFI for CD86 is shown for one experiment.
(TIF)

Figure S5 **Mature DCs are equally capable of OVA translocation into the cytosol than immature DCs.** Immature (UT) or matured DCs were incubated with biotinylated soluble OVA in the presence of the proteasome inhibitor lactacystin for 20min, washed and chased in the presence of lactacystin for another 40min. Cytoplasmic and membrane fractions were isolated, OVA was enriched through streptavidin pull-down and analyzed by WB. (A) OVA detected in cytoplasmic fractions or total cell lysates (TCL). (B) Purity control of starting material. Cytoplasmic and membrane factions were concentrated and analysed by WB for presence of the ER-marker grp94 or the lysosomal marker lamp2a. Although minor ER contamination could be detected in some cytosolic fractions, lysosomal markers were absent, suggesting that the cytoplasmic OVA band reflects true translocation rather than cross-contamination by OVA from endocytic organelles.
(TIF)

Methods S1 **OVA translocation assays.**
(DOCX)
