# Supplementary figures and images for: Intracellular Regulation of Cross-Presentation during Dendritic Cell Maturation
Source: PLoS One. 2013 Oct 3;8(10):e76801. doi: 10.1371/journal.pone.0076801 (PMC3789698; doi:10.1371/journal.pone.0076801)

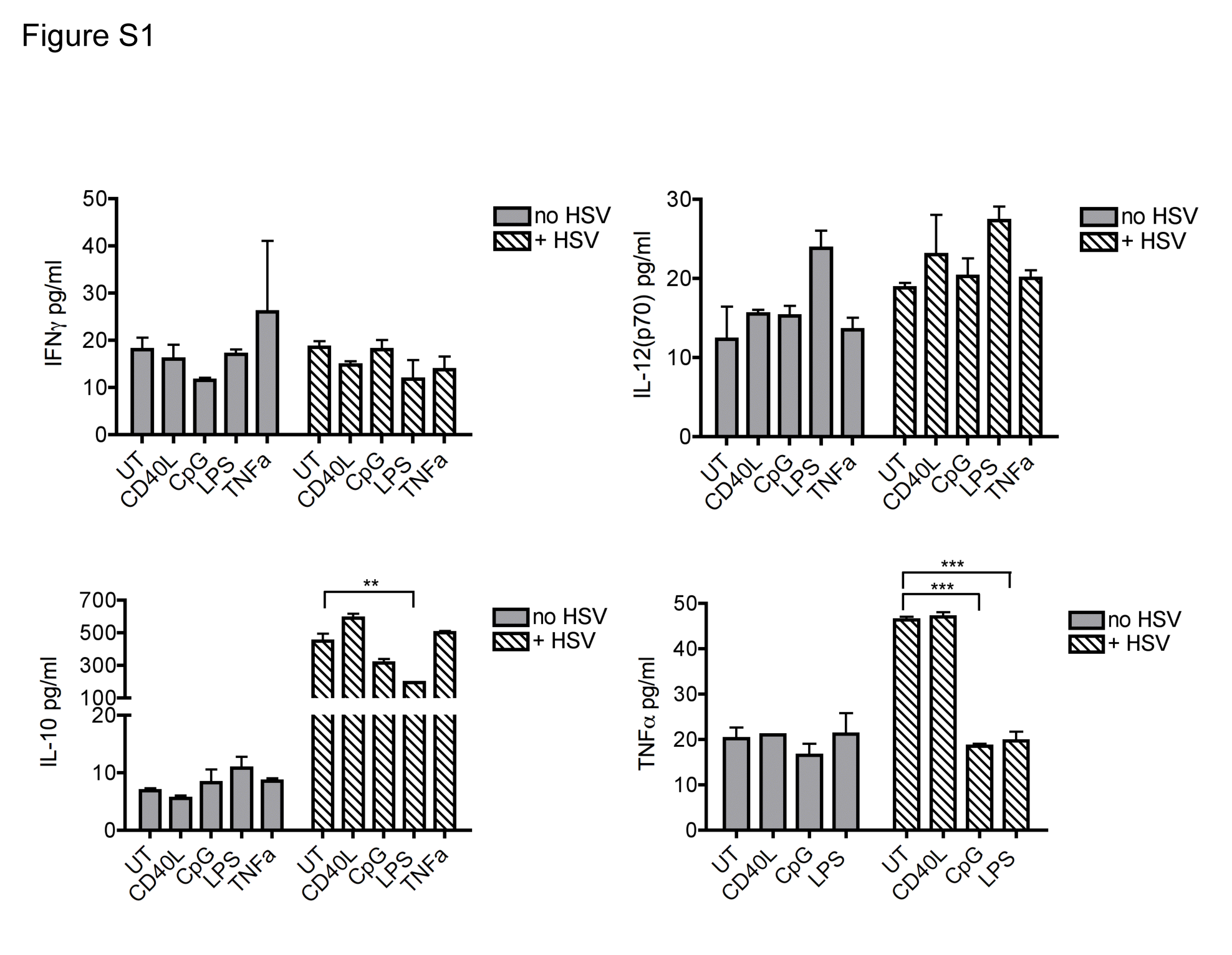

Supplement: Figure S1 — Cytokine secretion of immature and mature DCs with and without addition of HSV-infected HeLa cells. The cytokine profile of untreated or matured DCs was assessed for the indicated analytes with a magnetic bead-based assay. DCs were matured for 22h or left untreated, washed, and re-cultured with our without HSV-1 infected HeLa cells for another 20h prior to supernatant collection. Supernatants from 3 independent experiments were used. Error bars depict SD, ** p<0.01, *** p<0.001. (TIF) [file pone.0076801.s001.tif]

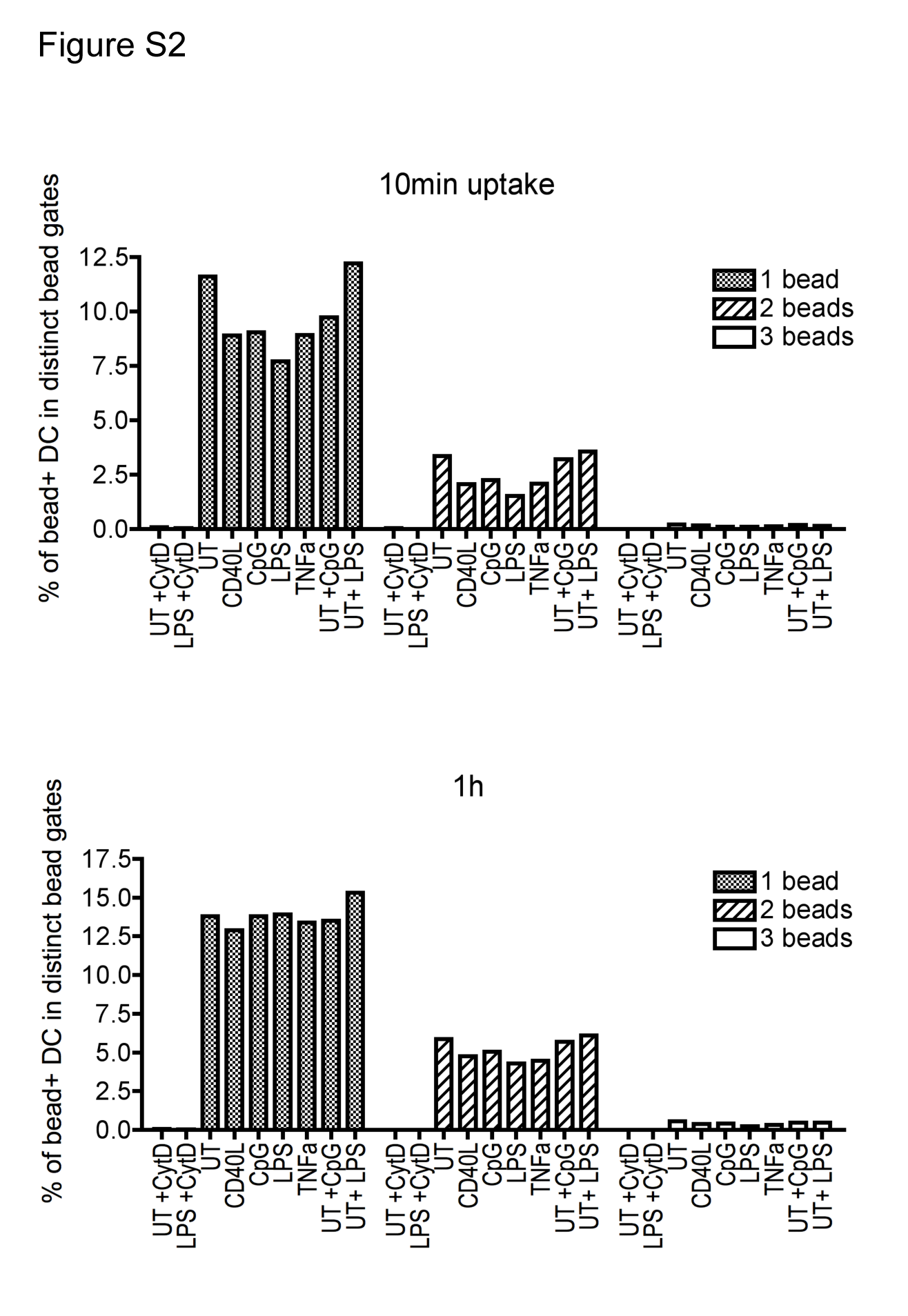

Supplement: Figure S2 — Uptake of OVA-coated YG beads. Mature and immature DCs were fed OVA coated beads as described in the methods section. For samples indicated with “+”, beads were added in combination with CpG, LPS or cytochalasin D. DCs with extracellular beads attached were excluded based on anti-OVA staining followed by a-rb A647. The percentage of cells containing either 1 bead, 2 beads or 3 beads after a 10min uptake (upper panel) or 1h uptake (lower panel) is shown. (TIF) [file pone.0076801.s002.tif]

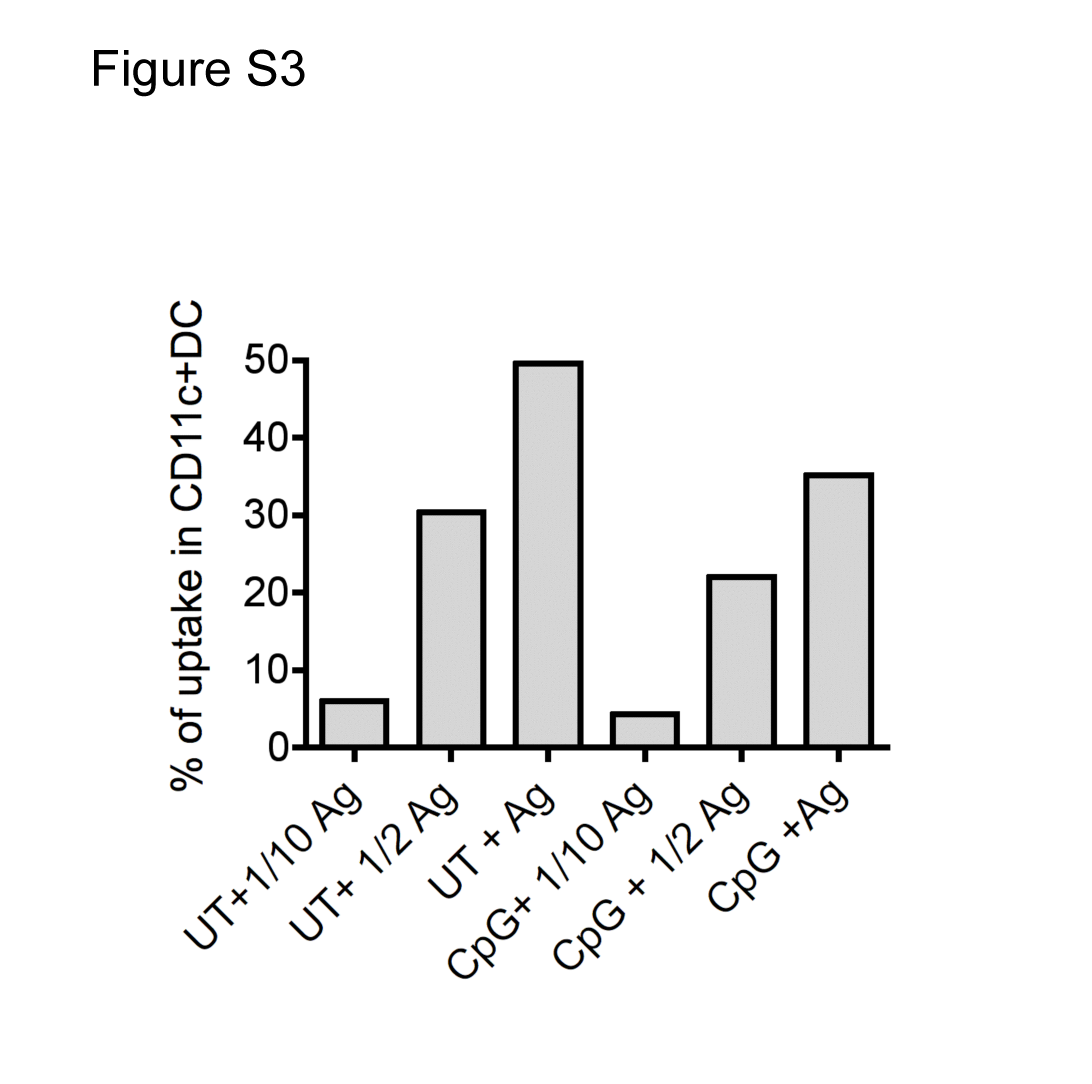

Supplement: Figure S3 — Antigen uptake titration in immature and CpG matured DCs. To identify a dose of antigen where immature DCs take up comparable amounts as matured DCs, DCs were prepared and pulsed with different amounts of CFSE-labeled necrotic HSV-infected HeLa cells in 96 wells as for antigen presentation assays. DCs were pulsed with usual amounts of HeLa-HSV (DC:HeLa ratio 4:1), half the amount (“1/2 Ag”) or one tenth (“1/10 Ag”). After 4h, uptake was assessed by flow cytometry. Percentage of uptake is calculated as CFSE+CD11c+ fraction of CD11c+ DCs. (TIF) [file pone.0076801.s003.tif]

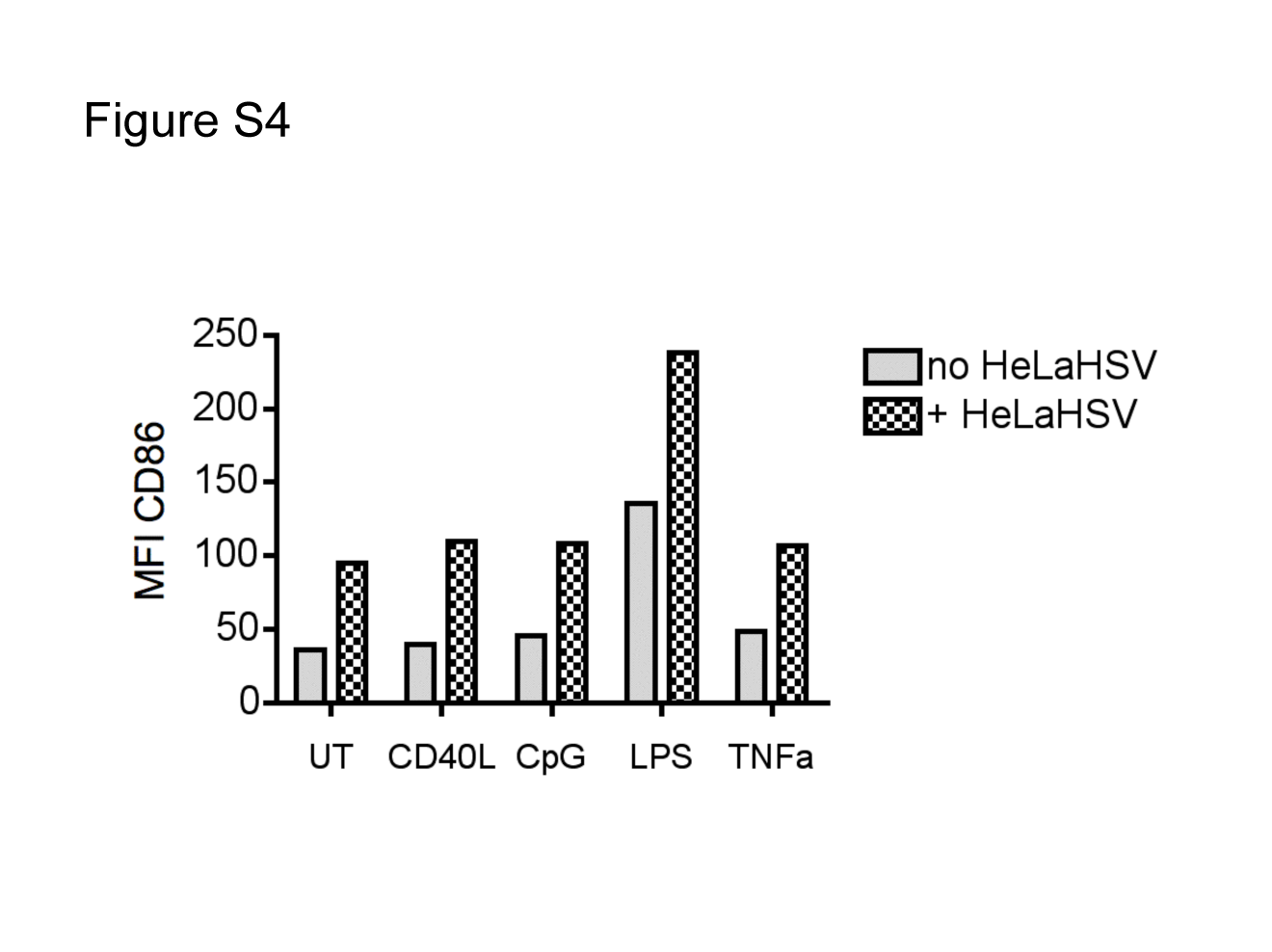

Supplement: Figure S4 — Matured DCs are not refractory to activating ligands in virally infected cells. Matured DCs up-regulate co-stimulatory molecules further after encounter of virally infected cells. Immature (UT) or matured DCs were cultured as for cross-presentation assays with HSV-infected HeLa cells. Phenotype was assessed prior and post co-culture with infected HeLa cells, MFI for CD86 is shown for one experiment. (TIF) [file pone.0076801.s004.tif]

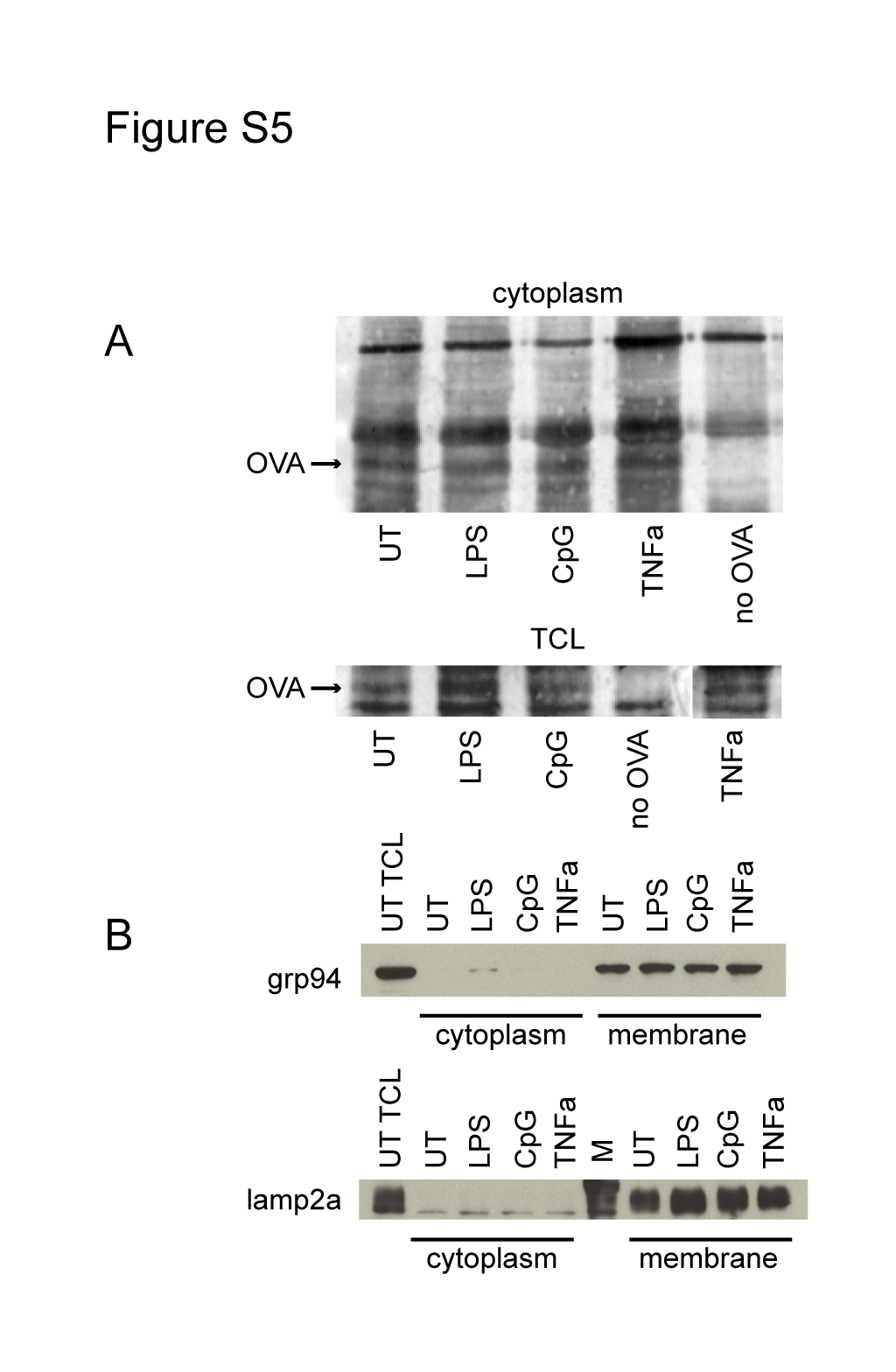

Supplement: Figure S5 — Mature DCs are equally capable of OVA translocation into the cytosol than immature DCs. Immature (UT) or matured DCs were incubated with biotinylated soluble OVA in the presence of the proteasome inhibitor lactacystin for 20min, washed and chased in the presence of lactacystin for another 40min. Cytoplasmic and membrane fractions were isolated, OVA was enriched through streptavidin pull-down and analyzed by WB. (A) OVA detected in cytoplasmic fractions or total cell lysates (TCL). (B) Purity control of starting material. Cytoplasmic and membrane factions were concentrated and analysed by WB for presence of the ER-marker grp94 or the lysosomal marker lamp2a. Although minor ER contamination could be detected in some cytosolic fractions, lysosomal markers were absent, suggesting that the cytoplasmic OVA band reflects true translocation rather than cross-contamination by OVA from endocytic organelles. (TIF) [file pone.0076801.s005.tif]
