# Supplementary material for: Intracellular Regulation of Cross-Presentation during Dendritic Cell Maturation
Source: PLoS One. 2013 Oct 3;8(10):e76801. doi: 10.1371/journal.pone.0076801 (PMC3789698; doi:10.1371/journal.pone.0076801)
Supplement: Methods S1 — OVA translocation assays. (DOCX) [file pone.0076801.s006.docx]

**Methods S1**

*OVA translocation assays*

OVA was biotinylated using Sulfo-NHS-LC-Biotin (Thermo Scientific). 12x10^6^ immature or mature DCs were allowed to take up soluble biotinylated OVA for 20min in the presence of 15μM lactacystin (EMD). To achieve equal uptake, immature DCs were incubated with 0.75mg/ml, TNFα- and CpG-treated DCs with 0.9mg/ml and LPS-treated DCs with 1mg/ml OVA. After washing, DCs were chased for an additional 40min in the presence of lactacystin. 2.5x10^6^ DCs were set aside for total cell lysate, prepared by extracting for 1h on ice in PBS containing 1% TritonX-100 and protease inhibitors (Roche). Cytoplasmic and membrane fractions were isolated with a subcellular proteome extraction kit (Calbiochem) and cytoplasmic OVA was enriched with streptavidin-agarose as described previously [[38](#_ENREF_38)]. After SDS-PAGE, OVA was detected by western blotting with rabbit anti-OVA serum (Polysciences) followed by HRP-conjugated goat anti-rabbit IgG. To control for their purity, cytoplasmic fractions were concentrated with a Microcon YM-30 centrifugal devices (Amicon) and prior to SDS-PAGE and blotting for Lamp2a (rabbit polyclonal antiserum, Abcam) and rabbit anti Grp94 (Enzo Life Sciences). Membrane fractions were used as positive controls for Lamp2a and GrP94.
